# Role of Hematopoietic Stem Cell Transplantation in Relapsed/Refractory Hodgkin Lymphoma

**DOI:** 10.4084/MJHID.2012.059

**Published:** 2012-10-02

**Authors:** Angelo Michele Carella

**Affiliations:** Direttore U.O. Ematologia, IRCCS San Martino, Genova

## Abstract

Hodgkin lymphoma is one of the most curable human tumors. Despite this, about 30% of these patients relapsed or are primary refractory to the first line treatment. Autografting is generally considered the standard of care for these patients. Alternative salvage strategies have been evaluated such as high dose sequential and tandem autografting strategies. In younger patients, refractory or early relapsed after autografting, allogeneic stem cell transplantation has been employed but this approach has been followed by significant concerns since the treatment related mortality, often exceeded 40–50%, and relapses were not uncommon. It is clear that patient selection remains an issue in all allografting reports.

At the end, new drugs and novel treatment strategies, that are based on our understanding of the disease biology and signaling pathways, are needed to improve treatment outcome for these patients. The two leading compounds Brentuximab Vedotin and Panobinostat, are currently under evaluation in several clinical trials.

## Introduction

Treatment outcome for patients with Hodgkin lymphoma has steadly improved over the last half-century. Today, about 70–80% of patients can be cured with modern treatment strategy. Unfortunately, some patients fail to attain an initial remission and approximately 30% of those, who achieve remission, relapse. Salvage chemotherapy followed by high-dose chemotherapy (HDC) and autologous stem cell transplantation (ASCT) has become the first choice in relapsed/refractory patients. Alternative strategies such as allografting, new cytostatic drugs and biological agents with proven efficacy in preclinical model may improve the outcome of relapsed/refractory patients to ASCT.

## Salvage Chemotherapy Treatments Before ASCT

The ideal salvage regimen should produce: a) high response rate, b) acceptable hematologic and nonhematologic toxicity and c) not impair the ability to mobilize peripheral blood stem cell mobilization. There is no consensus on the gold-standard of salvage chemotherapy. Regimens such as IGEV, DHAP, ICE or GDP are reasonable options but no direct comparison among them is available. In our opinion, patients who are non responders to platinum and/or gemcitabin containing regimens and have stable disease, can safely proceed to ASCT without any kind of further previous chemotherapy. An alternative salvage regimen could be used in patients with progressive disease and/or larger volume disease but the results are generally worse. It has recommended to use a salvage chemotherapy with which clinicians are confortable that results in acceptable toxicity, high response rates and good stem cell mobilization.

## Autologous Stem Cell Transplantation

At the end of ’80th, the Italian teams pioneered ASCT in 50 patients with advanced Hodgkin lymphoma.^1^ Soon after, a number of international Phase II clinical trials have confirmed the feasibility and the efficacy of this procedure alone. In all these retrospective studies, the results with ASCT appeared superior to those achieved with conventional salvage therapy alone. Unfortunately, the studies had a relatively small sample size and needed to be tested in controlled clinical trials to better clarify the role of ASCT. The first randomized trial comparing HDC/ASCT versus standard-dose salvage chemotherapy was carried out within the British National Lymphoma Investigation.^2^ As known, the study was stopped earlier because of an event-free survival advantage for the HDC arm (53% versus 10%; p=0.025). This study showed that HDC produced a better response rate, but, it did not answer the crucial question if was better to utilize ASCT in early or delayed phases of disease. The second controlled study was organized by the German Hodgkin Study Group (GHSG) in cooperation with our Unit in Genoa.^3^ They carried out a randomized trial in chemotherapy-sensitive patients comparing HDC (BEAM) vs conventional chemotherapy (two courses of Dexa-BEAM). At a median follow-up of 39 months, freedom from treatment failure was significantly better for the patients in the transplant arm (55% vs 34%, p=0.019) and, most importantly, the significant benefit from ASCT was confirmed in early and late first relapse. In UK and German trials, the overall survival did not differ in the two treatments. These results have been confirmed in other two case-control studies.^4^,^5^ The conclusion of all these trials was that HDC/ASCT is a superior treatment for patients with relapsed or refractory Hodgkin lymphoma and this approach has become the standard therapy for these patients.

In recent years, the ASCT techniques have been greatly improved, prognostic factors have been identified, and the optimal timing of ASCT has been evaluated. A new opportunity to further improve the ASCT procedure was to intensify the therapy with the use of augmented doses of mobilization regimens^6^ or additional therapy after PBSC collection.^7^ Both these techniques have been reported to be able to improve the outcome. The Cologne high-dose sequential (HDS) protocol utilized 2 cycles of conventional chemotherapy. The patients, who achieved a response, received high-dose cyclophosphamide with subsequent PBSC collection; soon after, high-dose methotrexate and etoposide and, finally, HDC (BEAM) and ASCT was given to the patients. The authors were able to demonstrate that HDS was feasible with acceptable toxicity.^8^ Recently, the German Hodgkin Study Group and EBMT reported the HD-R2 trial, a randomized comparison of high-dose sequential therapy followed by ASCT vs standard chemotherapy (DHAP) followed by ASCT. The median follow-up was of 42 months and no significant difference in freedom from therapy failure, progression free survival or overall survival was observed until now.^9^ In both randomized studies, the results in primary refractory patients were disappointing. Sweetenham et al. published a retrospective EBMT analysis in 175 patients who fail to enter remission with induction chemotherapy.^10^ The actuarial 5-year PFS and overall survival was 32% and 36%, respectively; moreover, patients receiving more than one chemotherapy line before ASCT did worse in terms of overall survival and progression-free survival. Similar results were achieved by GELTAMO Cooperative Group and Fermè in France in 62 and 157 patients, respectively.^11^,^12^ The results were better in the ABMTR analysis on 122 patients not responsive to first line therapy.^13^ The reasons of these discrepancies are not clear. Unfortunately, patients with rapidly progressive disease, poor performance status, older age, and poor stem cell harvest were not included in these reports; moreover, it is possible that these different results were subject to significant selection of patients.

## Can We Improve the Results after Autografting?

An intensification strategy has been tested with the use of double ASCT.^14^ There are limited registry data to support such a strategy. In the GELA multicenter H96 trial the patients were assigned to a single or double ASCT on the basis of presence of risk factors. The patients with primary refractory disease or with at least 2 poor risk factors, received double ASCT; all others received a single ASCT.^15^ The overall survival at 5 years was 46% in the poor risk patients with 6% transplant-related mortality. The IBMTR reported 21 Hodgkin lymphoma patients underwent a second ASCT.^16^ No difference was found in outcome; the results were particularly worse in patients receiving the second ASCT within 12 months from the first ASCT. Transplant-related mortality was 11% with the double ASCT and 5-year PFS was 30%. These trials demonstrated to be feasible but should be tested prospectively and compared with a standard single ASCT.

## Prognosis after Failure ASCT

The median overall survival for patients who progress or relapse after ASCT is 2 years or less. The GELTAMO cooperative group reported the long-term outcome in 175 patients who relapsed at a median time of 10 (range, 4–125) months after ASCT. One third and one fourth of them are alive and free of relapse at 3 years17. Adverse prognostic factors for progression-free survival were advanced stage at relapse and short time interval between ASCT and relapse less than 1 year. The patients who had both characteristics had 14% 3-year progression-free survival. Another analysis derived by Lymphoma Working Party of the EBMT database on 462 patients. After a median follow-up of 49 months, overall survival was 32% at 5-years. In multivariate analysis independent risk factors for overall survival were: advanced stage, poor performance status, age >50 years, early relapse. Overall survival was extremely low (12%) in patients with 2 or more risk factors while was high in 62% of patients with one factor only.^18^

## The Role of Functional Imaging Before ASCT

Retrospective studies suggest that Gallium or FDG-PET after salvage chemotherapy and before HDC/ASCT can be predictive of poor outcome.^19^ More recently, Moskowitz et al. have studied functional imaging after salvage chemotherapy reporting 75% 5-years event-free survival if functional imaging was negative and 31% for functional imaging-positive disease.^20^ Castagna et al. in 24 patients who underwent FDG-PET after 2 cycles of salvage chemotherapy, reported 2-years PFS of 93% for PET-negative and 10% for PET-positive patients.^21^ More recently, the role of PET has been analyzed in a group of 101 patients who received ASCT for Hodgkin lymphoma and non-Hodgkin lymphomas. Both FDG-PET after 2 courses of chemotherapy and clinical risk score were independent prognostic factors for progression-free survival after ASCT;^22^ moreover a poor outcome after ASCT was identified by the Houston team when PET resulted positive before ASCT.^23^ Despite these and other reports, PET scanning is still contradictory to predict outcome after ASCT. We need more robust results in order to determinate risk-adapted therapy outside of a clinical trial.

## Novel Agents

**Figure 1:**
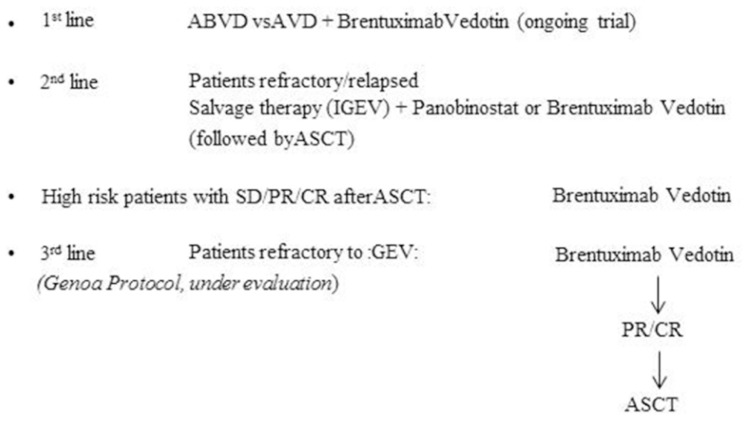
How to integrate targeted therapies into clinical practice for the treatment of Hodgkin Lymphoma.

After more than 30 years of absence of new drugs in this disease, in these last years several compounds have been identify as promising agents, mainly for the treatment of patients with relapsed HL. The two leading compounds, Brentuximab Vedotin and Panobinostat, are currently employed with excellent results. Brentuximab Vedotin has able to achieve 75% of overall response rate (34% CR) with a median duration of response for remitters patients of 25%.^24^ The potent pan-deacytilase inhibitor (Panobinostat) was able to determine an overall objective response of 27% of relapsed/refractory HL patients.^25^ Although the patients enrolled onto these studies had a poor prognosis and were previously highly pretreated with multiple chemotherapy regimens, almost all patients achieved tumor regression, mainly with Brentuximab Vedotin. A new strategy to improve autografting might be to incorporate these drugs with salvage regimen and/or as maintenance after stem cell transplantation. The new randomized trials with these drugs are ongoing and hopefully can modify the number of relapses. In figure 1 it has been showed the possible future perspectives employments of these drugs.

## Allogeneic Stem Cell Transplantation (AlloSCT)

Registry-based studies published in 1996 with myeloablative allografting gave disappointing results. AlloSCT was able to result in lower relapse rates but TRM exceeded 50% due to toxicity and infection complications. The explanation for this high mortality is uncertain but might include the selection of very high risk patients (many patients were allografted in advanced phase of the disease) combined with immunodeficiency, peculiar to Hodgkin lymphoma, leading to infectious complications. The use of low-dose preparative regimens, aimed at immunosuppression rather that tumor ablation, has decreased the early mortality rate in hematological neoplasias.^26^,^27^ This new approach have become increasingly popular after it became apparent that much of the anticancer effect from allografting is due to the adoptive immunotherapy. The largest cohort of patients (n=285) treated with RIC was recently reported by the Lymphoma Working Party of the EBMT.^28^ Patients have been highly pretreated with a median of 4 lines of therapy. The 100-day TRM was 12% but increased to 22% at 3 years. The best results were achieved in patients with chemosensitive disease. The development of chronic GVHD was associated with a higher TRM but a lower relapse rate. In a landmark analysis the development of GVHD by 9 months post-RIC was associated with lower relapse rate. Similar results were recently updated by MD Anderson Cancer Center in 58 patients with relapsed or refractory Hodgkin lymphoma.^19^ More recently, the final results of a multicenter phase II prospective study on the role of RIC were presented at ASH2010.^29^ Ninety-two patients with an HLA identical sibling or a MUD were treated with two courses of salvage chemotherapy. Seventy-eight patients (85%), who showed no progression, were eligible to receive a RIC. All patients with refractory disease died of lymphoma. Chronic GVHD was associated with a lower relapse incidence and a better progression-free survival. Patients allografted in remission had a significantly better outcome.

Sureda et al. has performed under Lymphoma Working Party of the EBMT the only analysis which to compare outcomes after RIC or myeloablative conditioning.^30^ Ninety-seven patients were allografte after RIC and 93 patients after myeloablative regimens. Non-relapse mortality was significantly decreased in the RIC group. Chronic GVHD significantly decreased the incidence of relapse, which translated into better progression-free survival and overall survival. The conclusion of this analysis was that the significative reduction of the transplant-related mortality after RIC was able to put in evidence the existence of a GVHL, which was able to improve the long-term outcome of relapsed and refractory Hodgkin lymphoma patients. The most direct evidence of a GVHL comes from Hodgkin lymphoma response to donor lymphocyte infusion. Peggs and associates described the results of RIC and donor lymphocyte infusion in patients with multiply relapses. The conditioning regimen included fludarabine, melphalan and alemtuzumab.^31^ Patients who had less than a CR or progression at 3 months received donor lymphocyte infusion. All patients engrafted and the 4-year progression free survival rate was 39% with a nonrelapsed mortality at 2 years of 16%. Sixteen patients received donor lymphocyte infusion at 3 months because of lack of response or progression and 8 of them achieved complete remission.

Other small series in Hodgkin lymphoma confirmed these results and reported response rate of 44–54% following DLI administration.^28^,^31^–^34^ All these data confirm the presence of a clinically effective GVHL effect.

## Tandem ASCT and RIC

Autografting and myeloablative allografting each offers potential roles in the treatment of relapsed/refractory Hodgkin lymphoma. Each modality has its limitations: ASCT has become the standard therapy for these patients but the relapses remain the most important cause of treatment failure; allografting is conditioned by high-mortality risks mediated by immunosuppressive drugs and GVHD. The safety of allografting has improved with the use of RIC, but, since graft vs Hodgkin lymphoma responses might be insufficient when Hodgkin lymphoma is bulky and tumor growth is rapid, it was thought that intensive lymphoma cytoreduction prior to RIC may allow reaction to be exploited.^34^ This tactic could provide the benefit of a conventional allograft but with a reduction in the typical acute toxicities and associate mortality of myeloablative therapy. Tandem ASCT/RIC was pioneered by the Genoa team in 10 patients with Hodgkin lymphoma and 5 patients with non-Hodgkin lymphoma.^34^ Thirteen of the 15 patients were considered to have poor prognoses due to chemotherapy-refractory disease (n=6), relapse (n=8), or bulky disease at ASCT. The allogeneic conditioning consisted of fludarabine and cyclophosphamide with short-course of methotrexate and cyclosporine post-grafting for GVHD prophylaxis. Nine of 12 patients in partial remission after ASCT achieved complete remission after RICT, while 3 patients had progressive disease. Donor lymphocyte infusions were given to 7 patients, in 2 patients for progressive or persistent disease and in 5 patients for mixed chimerism and persistent disease. At the time of the analysis, 10 patients were alive, while 5 patients died either from progressive disease (n=2), progressive disease and GVHD (n=1), chronic GVHD (n=1) or Aspergillus infection (n=1). The severity of GVHD appeared tolerable with only 1 patient dying directly from complications of extensive chronic GVHD. Recently, we have updated our results together with those of Humanitas Institute of Milan.^35^ A total of 27 patients underwent tandem ASCT/RIC. Twelve patients were chemosensitive and 15 chemorefractory at ASCT. The time interval between ASCT and RIC was 3 months. Chimerism studies indicated 100% donor-derived engraftment. Seven patients developed acute GVHD and 9 chronic GVHD (2 limited and 7 extensive). At the last follow up 17 patients (63%) were alive, 12 (70.6%) in CR and 5 (29.6%) with persistent disease. Ten patients expired (37%): 7 patients of disease progression, 1 patient of acute GVHD, 1 patient of extensive chronic GVHD and 1 patient of infection. Overall survival was 47 months at a median follow up of 46 months. These results suggest that GvLy may have a role on residual disease after ASCT. These results have been recently confirmed also by Gutterman et al. in 23 Hodgkin lymphoma patients. The U.K. Authors has employed a more complex approach with a combination of an intensive preparative regimen (BEAM) together with a profound T-cell depletion with Alemtuzumab as acute GVHD prophylaxis. They demonstrated that this approach was associated with sustained donor engraftment, high response rate, minimal TRD (7.6%), and a low incidence of extensive GVHD35. In conclusion, the tandem auto-allotransplant is a feasible approach in high-risk Hodgkin lymphoma and in the next future it should be evaluated on more patients, possibly in a randomized size.

## Conclusions

Stem cell transplantation is an effective approach in relapsed/refractory patients with Hodgkin lymphoma. Today the patients receive cell collected from peripheral blood in combination with growth factors and a rapid return of normal blood cells is achieved. The procedure is generally safe and, in some instances, can be carried out as an outpatient basis. A new strategy to improve autografting might be to incorporate the new drugs Panobinostat and Brentuximab Vedotin combined with salvage regimens and/or as maintenance after autografting.

The place of allografting in Hodgkin lymphoma remains a point for debate. Allografting has yielded lower relapse rates compared to ASCT, likely due to graft-versus-lymphoma effect. Long-term results of RIC demonstrate a progression free survival of 25–35% and an overall survival at 2–3 years of 35–60%. However, the long-term effects of acute and chronic graft-versus-host disease including both mortality and potentially serious morbidity represent a reason for caution in recommending this treatment approach. On this basis, we recommend RIC in the context of prospective clinical trials. In the next future we should try to reduce the relapse rates after RIC and prospectively compare RIC to both autografting preceeded and followed by Brentuximab Vedotin/Panobinostat.
